# Supplementary figures and images for: Pathogenic variants of sphingomyelin synthase SMS2 disrupt lipid landscapes in the secretory pathway
Source: eLife. 2022 Sep 14;11:e79278. doi: 10.7554/eLife.79278 (PMC9531943; doi:10.7554/eLife.79278)

$\alpha$ -SMS2

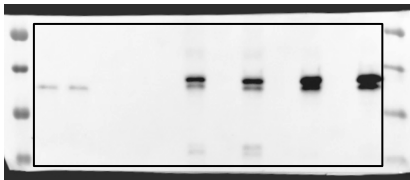

$\alpha$ -SMS2

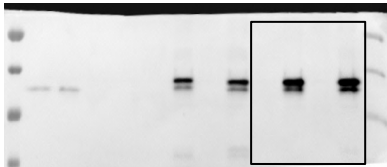

$\alpha$ -Na/K-ATPase

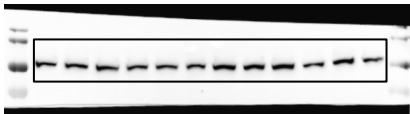

$\alpha$ -Na/K-ATPase

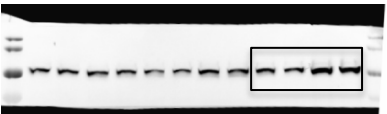

Supplement: Figure 2—source data 1. [file elife-79278-fig2-data1.pdf]

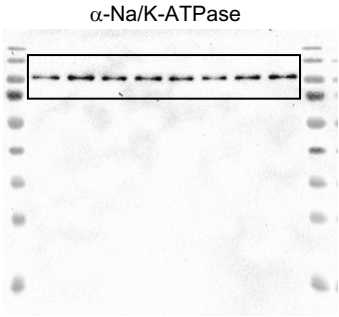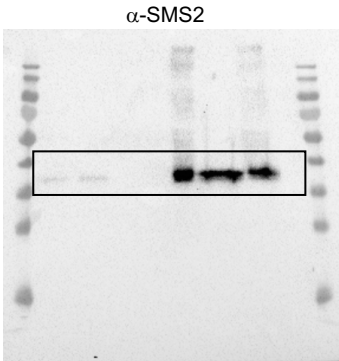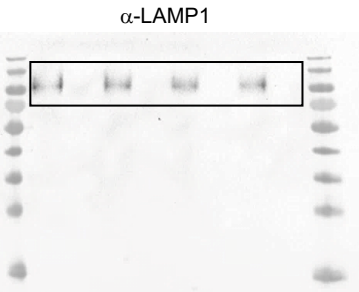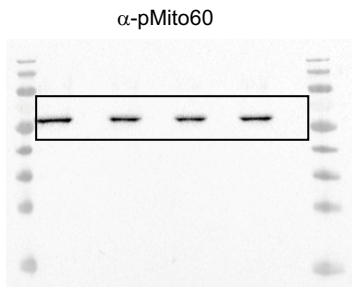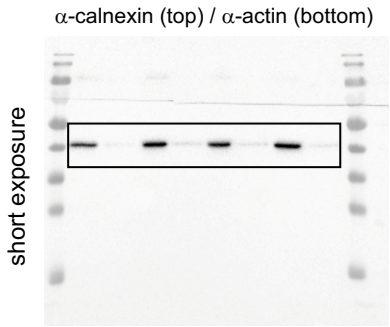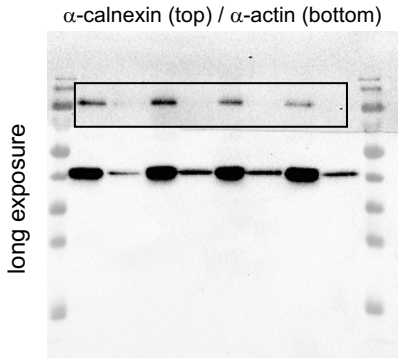

Supplement: Figure 4—source data 1. [file elife-79278-fig4-data1.pdf]
