# Supplementary material for: Pathogenic variants of sphingomyelin synthase SMS2 disrupt lipid landscapes in the secretory pathway
Source: eLife. 2022 Sep 14;11:e79278. doi: 10.7554/eLife.79278 (PMC9531943; doi:10.7554/eLife.79278)
Supplement: Figure 3—source data 1. [file elife-79278-fig3-data1.pdf]

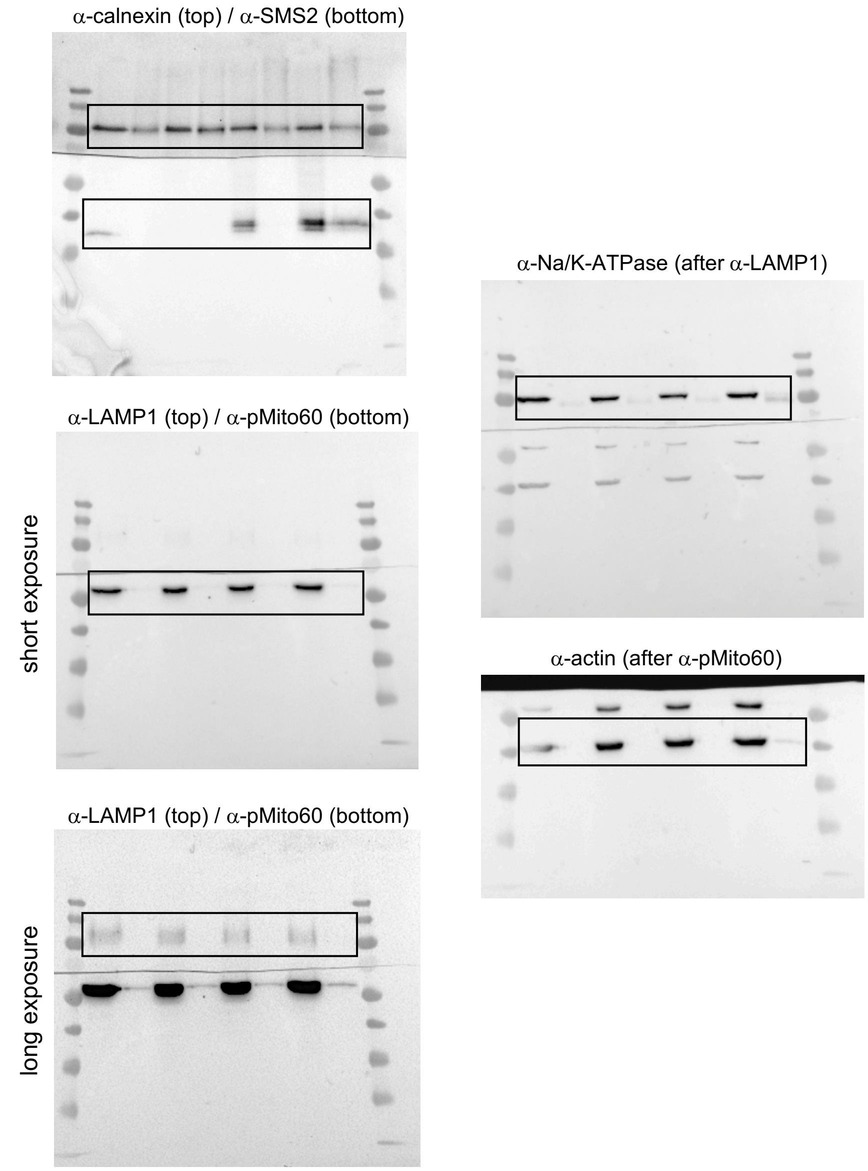

α-calnexin (top) / α-SMS2 (bottom)
α-LAMP1 (top) / α-pMito60 (bottom)
short exposure
α-LAMP1 (top) / α-pMito60 (bottom)
long exposure
α-Na/K-ATPase (after α-LAMP1)
α-actin (after α-pMito60)
